# Supplementary figures and images for: TRIP13 localizes to synapsed chromosomes and functions as a dosage-sensitive regulator of meiosis
Source: eLife. 2024 Aug 29;12:RP92195. doi: 10.7554/eLife.92195 (PMC11361706; doi:10.7554/eLife.92195)

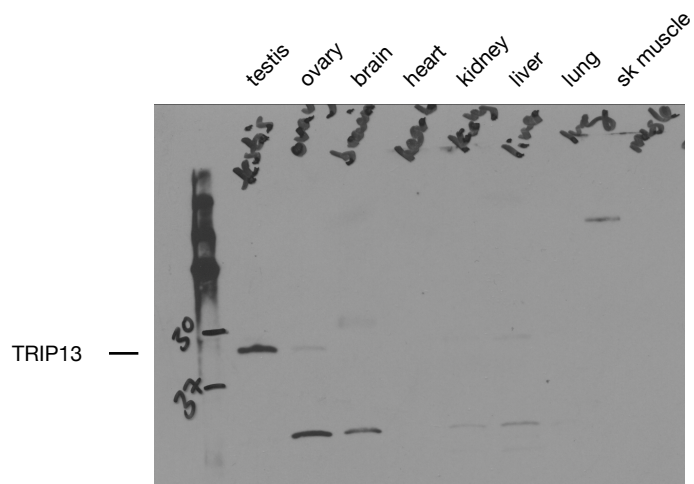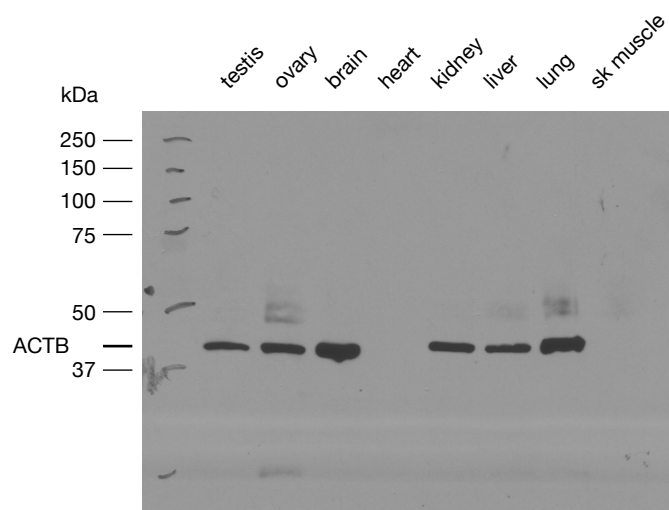

Supplement: Figure 1—source data 1. [file elife-92195-fig1-data1.pdf]

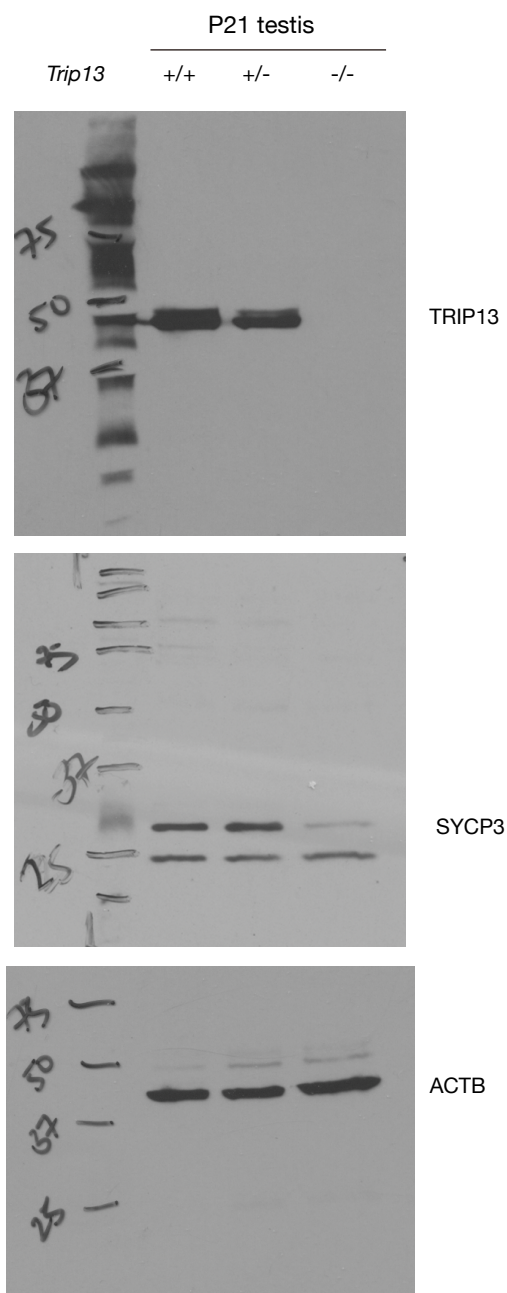

Supplement: Figure 2—source data 1. [file elife-92195-fig2-data1.pdf]

P20 testis

P20 testis

+/+  
+/3xFLAG-Trip13  
FLAG/FLAG

+/+  
+/Trip13-3xFLAG  
FLAG/FLAG

WB  
anti-FLAG

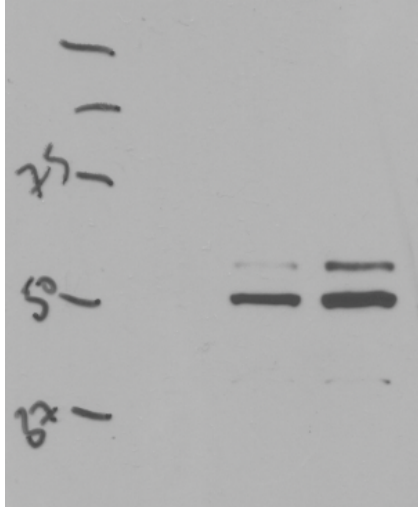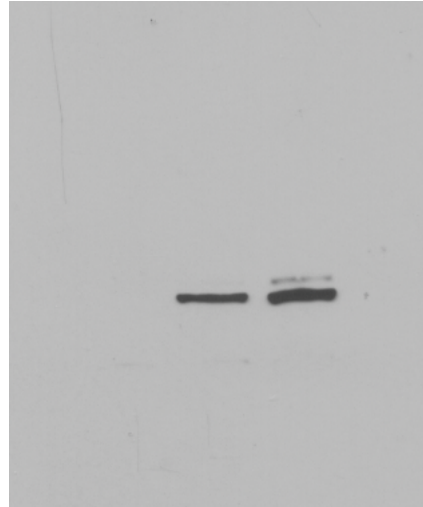

WB  
anti-TRIP13

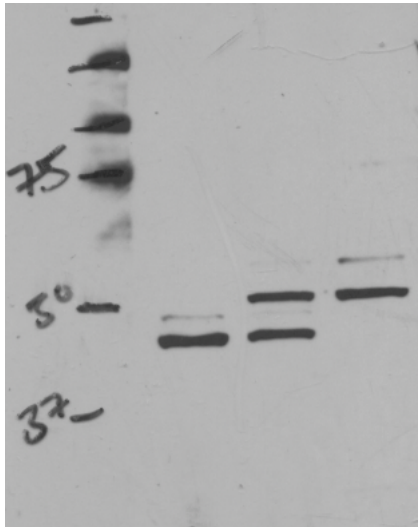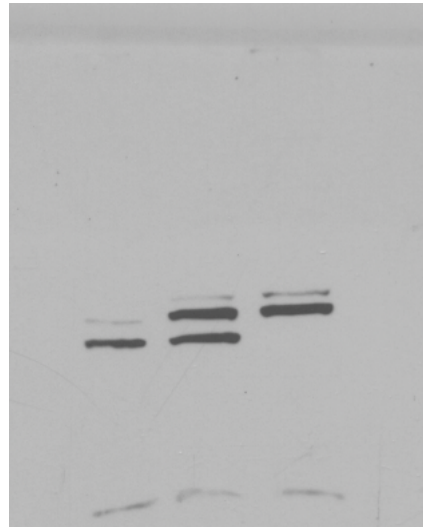

Supplement: Figure 7—source data 1. [file elife-92195-fig7-data1.pdf]
